# Aggregate distributional cost-effectiveness analysis: a novel tool for health economic evaluation to inform resource allocation

**DOI:** 10.1186/s41256-025-00415-z

**Published:** 2025-04-16

**Authors:** Shan Jiang, Boyang Li, Bonny Parkinson, Shunping Li, Yuanyuan Gu

**Affiliations:** 1 Macquarie University Centre for the Health Economy, Macquarie Business School and Australian Institute of Health Innovation, Macquarie University, Sydney, NSW Australia; 2 School of Political Science and Public Administration, Wuhan University, Wuhan, Hubei China; 3 Center for Health Management and Policy Research, School of Public Health, Cheeloo College of Medicine, Shandong University, Jinan, Shandong China; 4 NHC Key Lab of Health Economics and Policy Research, Shandong University, Jinan, Shandong China; 5 Center for Health Preference Research, Shandong University, Jinan, Shandong China

**Keywords:** Aggregate DCEA, Health inequality, Cost-effectiveness, Familial hypercholesterolemia screening, Health policy, Health equity

## Abstract

Health equity is a growing concern for policymakers across the globe. Conventional cost-effectiveness analysis (CEA), commonly used in evaluating health interventions, primarily focuses on the average and aggregate health outcomes in the targeted population, neglecting the distributional impacts on health equity. This gap calls for approaches that can quantify the impact of intervention of interest on health equity to support decision-making. Distributional Cost-Effectiveness Analysis (DCEA) offers a framework to assess the distributional impacts of health interventions. Based on DCEA, aggregate DCEA (A-DCEA) was proposed as a practical and simplified alternative to DCEA. Unlike full DCEA, which requires detailed subgroup data, A-DCEA utilizes aggregated data, making it accessible and feasible for broader use. In this commentary, we discuss the rationale for A-DCEA, outline the steps for its implementation, and highlight its applicability. The purpose of this article is to introduce A-DCEA as a pragmatic and accessible tool for evaluating the equity implications of healthcare interventions. A-DCEA can inform policymakers by incorporating equity considerations into healthcare decision-making, particularly when conducting a full DCEA is impractical due to data limitation. A-DCEA provides a valuable and accessible method for evaluating the distributional impact of interventions, promoting health equity in decision-making. Its adoption can lead to more informed health policy that considers health inequities as well as the efficient use of resources.

## Background

Health equity is an enduring challenge within healthcare systems worldwide [1]. Addressing disparities in health status across different socioeconomic groups has increasingly become a concern of healthcare policies, as health equity is an essential factor in achieving sustainable health improvements across populations [1, 2]. Countries such as England have specifically highlighted health equity as a policy concern, underscored by legislative instruments like the Health and Social Care Act 2012 [3]. This act mandates healthcare organizations to work toward reducing inequities in health outcomes, compelling policymakers to focus on interventions that are not only cost-effective but also equitable.

Conventional cost-effectiveness analysis (CEA) is the mainstay of health technology assessments conducted by agencies like the National Institute for Health and Care Excellence (NICE). CEA measures the efficiency of an intervention in terms of its cost per quality-adjusted life year (QALY) gained which is then compared to a cost-effectiveness threshold (representing either the maximum willingness to pay or the opportunity cost of healthcare resources) by policy makers to assess whether the intervention is ‘value for money’ or ‘cost-effective’. The key aim of CEA is the efficient use of healthcare resources. However, CEA only considers average health gains, overlooking who benefits most or least within the targeted population of interest. Consequently, this approach might inadvertently contribute to widening health disparities if an intervention that is cost-effective at the population level disproportionately benefits advantaged groups. The purpose of this commentary is to introduce Aggregate Distributional Cost-Effectiveness Analysis (A-DCEA) as a pragmatic and accessible tool for evaluating the equity implications of healthcare interventions. It outlines the methodology, discusses its practical benefits and limitations, and demonstrates its potential to support informed policy decisions by quantitatively addressing health equity alongside conventional cost-effectiveness measures.

## Aggregate distributional cost effectiveness analysis

Distributional cost-effectiveness analysis (DCEA), formally proposed by Asaria and his colleagues in 2015, extends traditional CEA by estimating the distribution of health effects across various social groups, such as by socioeconomic status or ethnicity [1, 4]. This allows healthcare policymakers to evaluate the equity implications of healthcare decisions quantitatively. However, conducting a full DCEA often requires substantial data on subgroup-specific parameters, which are not always available or feasible to collect.

Aggregate Distributional Cost-Effectiveness Analysis (A-DCEA) was recently developed by Griffin, Love-Koh, and their colleagues in 2018 as a more practical alternative to full DCEA [3, 5]. Full DCEA requires detailed subgroup data on costs, health benefits, and disease risk, which are often difficult to collect due to logistical, financial, or ethical constraints. For instance, obtaining subgroup-specific data for rare conditions can be especially challenging. In contrast, A-DCEA begins with the average benefit identified through CEA, scales it up based on the size of the targeted population under study, and then breaks down the aggregate benefits across different groups using the social patterns reflected in healthcare usage data for the specific disease being addressed [3]. This makes it a feasible option when detailed data are unavailable. The simplified nature of A-DCEA provides a way to approximate the distributional effects of health interventions, thus enabling equity considerations in decision-making without the intensive data requirements of full DCEA.

Currently, A-DCEA has not been extensively applied in health economic evaluation for resource allocation decision-making. The first application was by Love-Koh, who evaluated the 27 interventions using data on health benefits, costs, and patient population obtained from NICE website [3]. More recently, Meunier conducted two A-DCEA studies in collaboration with Griffin and Love-Koh in 2023 and 2024, focusing on treatments for diabetic macular oedema and non-small cell lung cancer, respectively. [6, 7] To the best of our knowledge, Wang’s article examining the distributional impact of familial hypercholesterolemia (FH) screening is the most recent application of A-DCEA and the fourth globally [8].

**Fig. 1:**
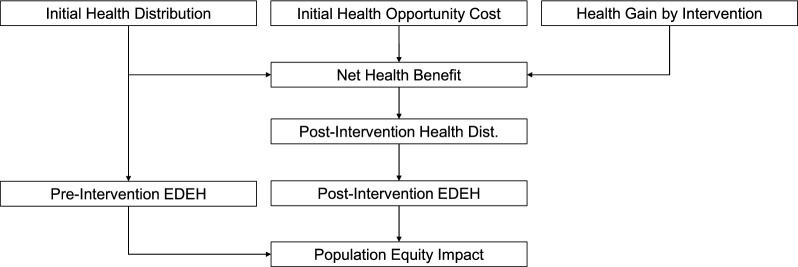
A-DCEA workflow. *Note*: Dist, distribution; EDEH, equally distributed equivalent health

The six-step framework for conducting A-DCEA, as outlined by Wang et al., offers a structured way to estimate the distributional impact of health interventions [8]. Their study synthesized economic evidence on population-wide screening for FH and utilized A-DCEA to assess the distributional impact of FH screening across various contexts. While the original article provides a more comprehensive explanation, complete with equations and in-depth methodologies, we provide a concise summary of the process below, accompanied by an explicit workflow chart to illustrate the steps involved (Fig. 1).

The process begins by estimating the baseline distribution of health across different population groups using quality-adjusted life expectancy (QALE) data, which helps identify existing health disparities before the intervention. Second, the distribution of health opportunity costs is applied across population groups to account for the health benefits forgone by reallocating healthcare resources. Later, the net health benefit (NHB) for each group is calculated by subtracting the opportunity costs from the health gains achieved through the intervention, allowing an understanding of the net impact on different subgroups.

Next, the NHB is added to the initial health distribution to obtain the post-intervention health distribution, providing insight into how the intervention alters health outcomes among groups. Subsequently, the Atkinson inequality index (or other inequality index such as Kolm index) is used to calculate the equally distributed equivalent health (EDEH) before and after the intervention, to assess its impact on health equity. Finally, comparing the pre- and post-intervention EDEH determines whether the intervention has reduced or exacerbated health inequality, with a positive outcome indicating a reduction in inequality.

## Policy implications for resource allocation

A-DCEA has significant value in informing health policy, particularly in contexts where data availability is limited. By providing an approximation of the distributional impact of health interventions, A-DCEA allows policymakers to incorporate equity considerations into decision-making processes without the need for extensive subgroup data. This capability makes it especially useful for national health systems and authorities (e.g., Medicare Benefits Schedule in Australia and National Administration of Health Security in China), where resource allocation considers both efficiency and equity objectives. Using tools such as the health equity impact plane, policymakers can visualize both the overall health impact and its distributional consequences [5]. This dual perspective is crucial for making informed decisions that do not inadvertently worsen health disparities. Moreover, A-DCEA's use of social welfare indices, such as the Atkinson or Kolm index, provides a means to quantify societal preferences for reducing inequalities, offering a more holistic view of the value of health interventions.

Alongside efforts to enhance the methodological framework of CEA, such as adopting a societal perspective, employing advanced dynamic models, and accounting for productivity losses and future medical costs [9–11], A-DCEA is driving the evolution of economic evaluation. It is a powerful tool for providing equity-focused insights to policymakers, particularly at a time when new and increasingly expensive health technologies are proliferating, necessitating assessments of both their cost-effectiveness and their distributional impacts. For example, the increasing use of genome sequencing in newborn screening and intensive care offers a comprehensive means to detect a wide array of congenital disorders [12, 13]. However, if access to genome sequencing varies by socioeconomic status, the technology’s impact on health equity remains uncertain. Another example is breast cancer screening, where the application of genome sequencing for screening is rapidly increasing [14], while disparities in the uptake rate of screening programs across ethnic groups are well-documented [15]. Failing to account for this in screening program evaluations risks overlooking the fact that some women may not benefit from the intervention, potentially exacerbating health inequity.

It is crucial to inform decision-makers about the distributional impacts of interventions and to use quantitative evidence to demonstrate that an intervention may be more favourable if the most deprived groups gain proportionally greater health benefits, thereby reducing overall health inequity. This was clearly shown in Wang et al.'s assessment of the distributional impact of FH screening [8]. These insights are essential for ensuring that health interventions not only improve health outcomes but also align with broader equity goals, particularly in public health contexts where reducing disparities is a priority.

## Limitations of A-DCEA approach

The A-DCEA approach has notable limitations inherent in its methodology. First, the A-DCEA framework typically applies disease-specific healthcare utilisation parameters uniformly across both advantaged and disadvantaged populations. This approach fails to account for potential social variations in technology utilisation patterns. For instance, A-DCEA often assumes equitable access to new technologies across all groups, disregarding the possibility of disparities in uptake. Such disparities are likely to arise due to varying levels of barriers linked to the complexity of healthcare systems. Advantaged groups generally enjoy better access to healthcare systems and new technologies, while disadvantaged populations face more significant barriers, resulting in worse access. By assuming equal access, A-DCEA may overestimate the extent to which a new technology could reduce health inequalities. Another critical limitation relates to the assumption of uniform incremental QALY gains across social groups. A-DCEA analyses typically presume that disadvantaged and advantaged groups experience identical QALY benefits from new interventions. This oversimplification risks overstating the potential reductions in health inequality, as socially advantaged groups are more likely to achieve greater benefits due to factors such as higher treatment adherence and fewer comorbid conditions. Consequently, this assumption may exaggerate the impact of new technologies on reducing health disparities. Additionally, for the sake of simplicity, A-DCEA generally does not account for uncertainties surrounding key distributional parameters, such as the baseline health distribution of different groups or the distribution of opportunity costs across these groups. This omission constrains the ability to fully characterise the uncertainty inherent in funding decisions, limiting the robustness of the conclusions drawn.

## Conclusions

This commentary has introduced A-DCEA as a pragmatic method to incorporate equity considerations into health economic evaluations, especially when detailed subgroup data are unavailable. It has outlined the methodology, discussed the benefits and limitations of the A-DCEA framework, and highlighted its value through recent applications, such as familial hypercholesterolemia screening. A-DCEA offers a simplified yet effective means of assessing the distributional impacts of health interventions, enabling equity considerations to be embedded into decision-making processes. By providing a way to estimate health gains across different population groups using readily available data, A-DCEA fills a critical gap in conventional CEA especially when data are limited such as in low- and middle-income countries. This approach has the potential to make health policy more equitable, contributing to a reduction in health inequalities that persist across socioeconomic strata. Wider adoption of A-DCEA by researchers and policymakers could ensure that equity becomes a cornerstone of health intervention assessments, ultimately leading to fairer health outcomes for all.

## Data Availability

Not applicable.

## Abbreviations

CEA: Cost-effectiveness analysis
DCEA: Distributional cost-effectiveness analysis
FH: Familial hypercholesterolemia
ICER: Incremental cost-effectiveness ratio
IMD: Index of multiple deprivation
NHB: Net health benefit
NICE: National institute for health and care excellence
QALY: Quality-adjusted life year
QALE: Quality-adjusted life expectancy
EDEH: Equally distributed equivalent health
